# The Relationship Between Negative Self-Concept, Trauma, and Maltreatment in Children and Adolescents: A Meta-Analysis

**DOI:** 10.1007/s10567-024-00472-9

**Published:** 2024-02-22

**Authors:** Daniela M. Melamed, Jessica Botting, Katie Lofthouse, Laura Pass, Richard Meiser-Stedman

**Affiliations:** Department of Clinical Psychology and Psychological Therapies, University of East Anglia, Norwich Research Park, Norwich, Norfolk, NR4 7TJ UK

**Keywords:** Trauma, Maltreatment, Negative self-concept, Children, Adolescents

## Abstract

Experiencing trauma in childhood is a global public health issue linked to worse physical and mental health outcomes, including Post-Traumatic Stress Disorder (PTSD). Self-concept is a transdiagnostic concept linked to various psychopathologies and understanding its unique relationship to trauma is important. This meta-analysis aimed to understand the size of the effect between trauma and maltreatment and self-concept in children and adolescents. The current meta-analysis searched PubMed, PILOTS, PsycINFO, and Web of Science databases. Inclusion criteria involved studies with defined trauma exposure, valid measures of self-concept, and participants’ mean age under 18 years old. One-hundred-and-thirty-four studies were included in the meta-analysis (N = 255,334). A random-effects meta-analysis was performed. A small negative relationship was observed between trauma exposure and self-concept (r = − 0.20, 95% CI − 0.22, − 0.18). This relationship was significantly moderated by some variables (type and nature of trauma exposure) but not others (participant gender, type of self-concept measure, quality of studies, country economic status). A small relationship between trauma exposure and negative self-concept in children and adolescents was detected, with repeated trauma exposure and type of trauma exposure moderating this relationship. This provides important directions for clinical practice around providing support for those exposed or most vulnerable to experiencing trauma.

**Supplementary Information:**

The online version contains supplementary material available at 10.1007/s10567-024-00472-9.

## Introduction

Exposure to child trauma or maltreatment is an issue that impacts children worldwide. Childhood maltreatment can refer to any type of abuse that has the potential to cause harm to individuals (Gardner et al., 2019; Krug et al., 2002); this can include physical, emotional and sexual abuse, and neglect. Approximately 36%, 22%, and 16% of children have experienced emotional abuse, physical abuse, and neglect, respectively, worldwide (World Health Organization, 2014). The prevalence of poly-victimization has been reported to be between 38% in children from Low–Middle-Income Countries (Le et al., 2016). The prevalence of certain types of maltreatment, such as sexual abuse, is reported to be higher among girls compared to boys (Stoltenborgh et al., 2011). Many studies have explored the long-term effects of exposure to childhood trauma and maltreatment. Meta-analyses looking at the impact of childhood maltreatment in adulthood have found that experiencing maltreatment significantly increases the risk of developing chronic illnesses (Nelson et al., 2017) and other physical health outcomes, such as obesity and persistent physical symptoms (Afari et al., 2014; Danese & Tan, 2014) over the lifespan.

Other studies have found that having experienced physical and sexual abuse significantly increases the risk of receiving a diagnosis of anxiety and depressive disorders in adulthood (Gardner et al., 2019). This association is reported to be larger for women than for men (Gallo et al., 2018). The association between exposure to childhood trauma and mental health outcomes is found to be larger in those with greater exposure, with a particularly increased risk for those exposed to emotional abuse and neglect (Humphreys et al., 2020). Additionally, those who experienced physical and sexual abuse in childhood have an over 70% increased risk of drug abuse in adulthood, with women additionally being at a greater risk than men (Halpern et al., 2018). Therefore, a strong link exists between exposure to trauma in childhood and later life mental and physical outcomes, with greater exposure to trauma and being female increasing this risk.

There is additionally a body of literature that has explored this relationship between exposure to trauma and mental and physical health in childhood. Evans et al.’s (2008) meta-analysis found a medium effect between children’s exposure to domestic violence and post-traumatic stress symptoms, internalizing behaviors, such as anxiety, and externalizing behaviors, such as aggression in childhood. Other studies have found that exposure to childhood trauma is linked to poor educational outcomes (Romano et al., 2015) and many studies have found that victimization in adolescence has been linked to increased post-traumatic stress symptoms (Soler, 2012) and linked to a decrease in self-compassion (Tanaka et al., 2011). Research has found that up to 25% of children exposed to a traumatic event met threshold for a diagnosis of post-traumatic stress disorder, with rates increased for children exposed to interpersonal traumas (Alisic et al., 2014; Peltonen & Punamaki, 2010; Punamaki, 2008; Taylor & Chemto, 2004).

A particular outcome that has been explored in the literature is the relationship between exposure to childhood trauma and self-concept. The term self-concept is an umbrella term which refers to a collection of beliefs, ideas, and perceptions about oneself; it refers to one’s own self-image (Burnett, 1994). This umbrella term encompasses concepts, such as self-esteem and self-identity. Self-concept is additionally an important transdiagnostic concept in mental health, often linked to various psychopathologies (Zeigler-Hill, 2011). According to the International Classification of Diseases-11th version (ICD-11) (WHO, 2018), negative self-concept encompasses one of the three additional criteria needed to meet the diagnosis of Complex Post-Traumatic Stress Disorder (PTSD). The ICD-11 more specifically refers to this concept as beliefs about oneself as being worthless or diminished with accompanying feelings of guilt or shame (WHO, 2018). It has been widely studied in the literature and most notably Rosenberg’s Self-Esteem Scale (1965) has been used internationally to measure this.

Longitudinal and retrospective studies have looked at this relationship between self-concept and exposure to traumatic events more specifically. Research suggests that exposure to traumatic events in an individual’s early years has an impact on one’s sense of self (Silvern et al., 1995). As noted earlier, exposure to childhood trauma has been linked to various psychopathologies and poor outcomes later in life. Many studies have evaluated the relationship between exposure to childhood trauma and self-esteem retrospectively (Kuo et al., 2012; Luszczynska et al., 2009). Some studies have explored self-concept as a moderator between trauma exposure and mental health outcomes, where self-concept has been found to significantly moderate the relationship between trauma exposure and PTSD (Salami, 2010). Cognitive theories of PTSD that trauma-related appraisals play a major role in the emergence of this disorder (Ehlers & Clark, 2000); a number of papers suggest that when appraisal relate to the self this effect is particularly strong in adults (Gomez de la Cuesta et al., 2019). Pacheco’s (2014) systematic review investigated the effect of child maltreatment on school performance, peer relationships, social competence, and self-esteem in both adults and children and found that exposure to trauma increased difficulties in all of those areas. However, to the authors’ knowledge, there is no published meta-analysis that specifically explores the size of the relationship between exposure to trauma and self-concept in children and adolescents. Given previous research that has suggested that the effect of trauma exposure on well-being is larger for women than for men and is larger with increased trauma exposure and different types of trauma exposure (Gallo et al., 2018; Humphreys et al., 2020), it is important to study these potentially moderating effects in children and adolescents. Other potential moderators also warrant attention. Exposure to traumatic events is experienced by children worldwide and further understanding of the extent to which country status may also moderate this relationship is important to explore. Some theorists have suggested that the processing of traumatic experiences is shaped by cultural factors, e.g., in more collectivist cultures self-concept is based less on autonomy and uniqueness (Jobson et al., 2014). The age at which children and adolescents experience traumatic events may also be pertinent, as multiple cognitive developmental processes may shape how traumatic experiences are experienced and stored in autobiographical memory (Salmon & Bryant, 2002).

## Aim of Meta-analysis

Therefore, the aim of the current review was to systemically examine and meta-analyze studies to explore the relationship between exposure to traumatic events and maltreatment and its association with self-concept in children and adolescents. For this review, we used the DSM-5 PTSD (APA, 2013) definition of a traumatic event (i.e., an event that involved “actual or threatened death, serious injury, or sexual violence”) and considered maltreatment to be any form of sexual abuse, physical abuse, emotional abuse, or neglect. We considered peer victimization to be a form of traumatic event (given the physical threat involved).

The main research questions are as follows:

i. What is the size of the effect of the relationship between negative self-concept and exposure to trauma and maltreatment in children and adolescents?
ii. What factors moderate this relationship specifically?

## Methods

### Registration

The current meta-analysis was prospectively registered with PROSPERO on October 12th, 2020 (CRD42020200148). No similar research protocols were identified through PROSPERO and to the authors’ knowledge no previous meta-analysis was published on this topic. The PROSPERO protocol included an additional research question looking at the relationship between exposure to traumatic events and mental health in only trauma-exposed children and adolescents. The current review solely focuses on question one due to the large amount of studies included in the final review; the second question will be explored separately.

### Selection of Studies

Studies were selected following a systematic search for relevant publications from 1980 (when PTSD was first introduced in the DSM) in PubMed, PILOTS (International Literature on Traumatic Stress; US Department of Veterans Affairs, 2015), PsycINFO, and Web of Science to the 31st of October 2020, when the search was completed; searches were then updated on the September 18th, 2023. The following search terms were used in the study to answer the research questions: adolescent* OR child* OR teen*” AND “physical abuse” OR “sexual abuse” OR neglect OR “emotional abuse” OR maltreatment* OR trauma AND “self esteem” OR “self-concept” OR “sense of self” OR “self perception” OR “self worth.”

### Inclusion and Exclusion Criteria

To be included in the analysis, studies were required to meet all the following inclusion criteria: participants mean age was less than 18 years old; the study included a measure of trauma exposure or there was a defined trauma-exposed group and non-exposed group; and outcomes were reported on a validated measure of self-concept. For the purpose of this study, exposure to a traumatic event was defined using DSM-5 (APA, 2013) criteria, while maltreatment was considered to mean exposure to any form of sexual abuse, physical abuse, emotional abuse, or neglect. Studies were excluded if they only provided qualitative data on self-concept, if they were not in English, or if the mean age was above 18 years old. We also excluded studies for the following reasons which were determined after the review was registered on PROSPERO: those that included orphan status, if participants came from a sample which was selected for having a mental health difficulty, was an at-risk sample or had a substance abuse problem, or if the self-concept measure used did not measure self-worth. These were not planned exclusion criteria but when screening studies it was decided to exclude these post hoc.

### Screening Method

**Fig. 1:**
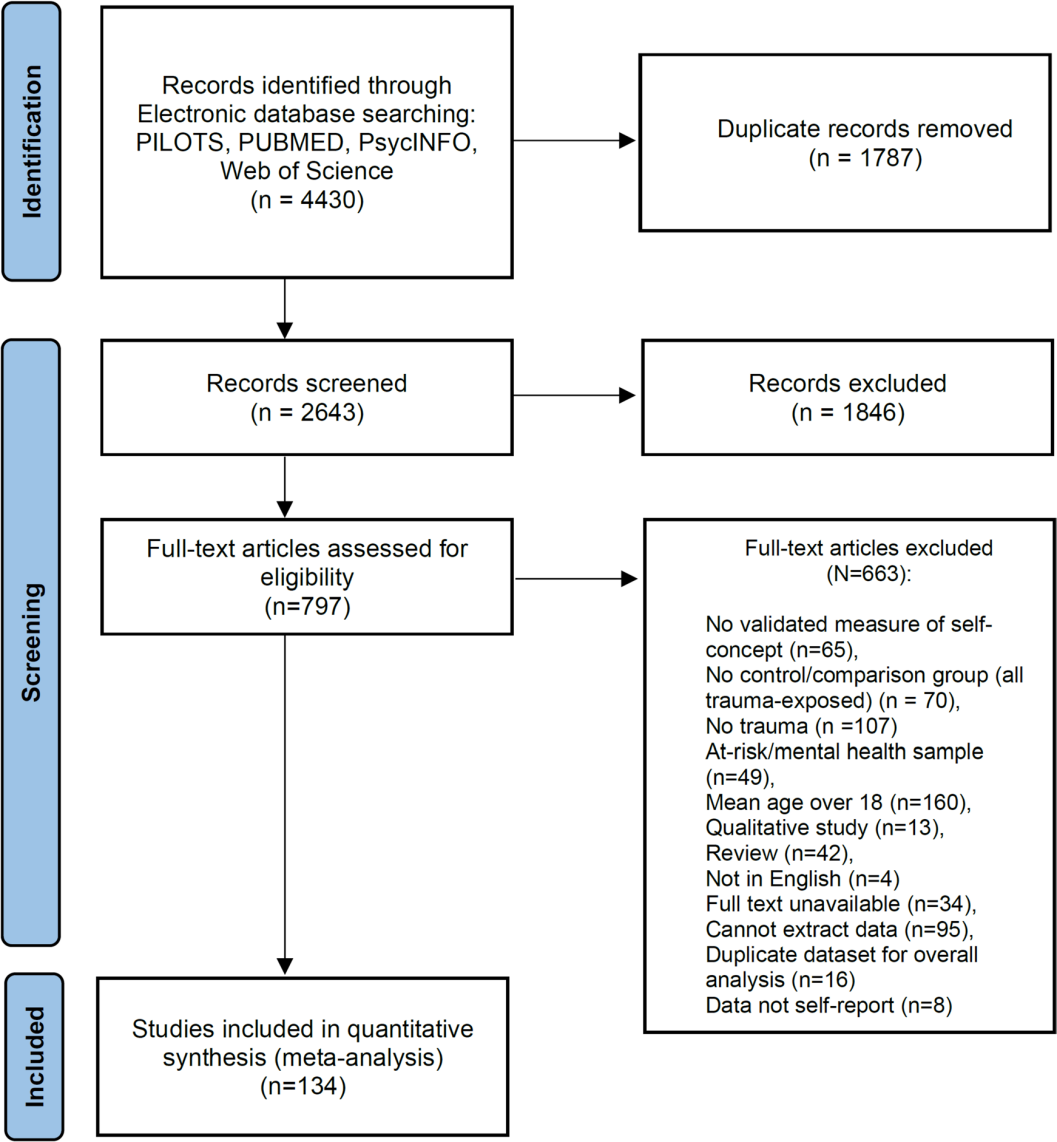
PRISMA diagram of screening methods

The process for selection of peer-reviewed articles were conducted according to the Preferred Reporting Items of Systematic Reviews and Meta-Analyses (PRISMA) guidelines (Moher et al., 2009) and is outlined in the PRISMA diagram in Fig. 1.

### Data Extraction

A stepwise approach was used to identify the studies that met inclusion criteria. Duplicate records were identified and removed by the first author. Titles and abstracts identified in the search were initially screened using the above inclusion and exclusion criteria. This was done to exclude articles that were not relevant to the question. Titles and abstracts of the excluded articles were reviewed to ensure these were appropriately excluded. Full texts were screened by two independent researchers. This was done systematically where questions and difference in scores were discussed until consensus was reached.

A data extraction spreadsheet was used to collate the following information from each study: type of study design, total number of participants, mean age of study participants, type of trauma exposure, participant characteristics (e.g., gender, age, and socioeconomic status), information on the measures used, mean scores and standard deviations on measures, and/or relevant statistics reported on the relationship between the variables of interest. Data were extracted by one author and checked by another author.

### Quality Assessment

Quality assessment was rated using an adapted version of the STROBE Statement: Checklist of items that should be included in reports of cross-sectional studies (Von Elm et al., 2014) to fit the research question (see Supplementary Material 1). Each study could be awarded a maximum of 14 points. Studies that had less than four points were labeled as “low quality,” studies that had between four and nine points were labeled as “medium quality,” and studies that had over nine points were labeled as “high quality.” Quality ratings for each study are reported in Supplementary Material 2. The first author rated each of the studies and an independent rater assessed 43% of studies. Inter-rater reliability was assessed using Single Score Intraclass Correlation Coefficients and 95% intervals. There was moderate moderator agreement (McHugh, 2012) between the raters (ICC = 0.71, 95% CI 0.56, 0.81).

### Data Synthesis

A random-effects meta-analysis was conducted using metafor in R (Viechtbauer, 2010). Pearson’s correlation coefficient r was used as the effect size of interest for the current meta-analysis as it is most easily interpretable (Field, 2001). This correlation was the most common statistic reported across the various studies. Studies that reported other statistics such as p-values and t tests could be converted to an ‘r’ effect size. Correlation coefficients were pooled where there were multiple outcomes for each study. The 95% confidence intervals (CI) for each outcome were used to demonstrate the certainty of results. Effect sizes were combined using Fisher’s Z transformation; this method used a weighted average to take into account differing sample sizes (Borenstein et al., 2009). The presence of heterogeneity was determined using Cochran’s Q; the *I*^2^ statistic (i.e., the percentage of variation across studies that is due to heterogeneity rather than chance; Higgins & Thompson, 2002) was also reported, and prediction intervals were calculated to indicate the expected range of true effects in future similar studies (IntHout et al., 2016).

### Moderator Analysis

It was decided when planning the review that moderator analyses would be undertaken if ten studies or more were identified in order to examine possible sources of heterogeneity. Variables were identified a priori to examine as study-level moderator variables. The metafor package uses mixed-effects meta-regression models to evaluate the role of moderator variables, where a moderation effect is observed if an omnibus test is significant; p-values for these tests are reported in the Results.

Several moderator analyses were conducted to address theoretical questions: whether the type of trauma was sexual abuse only or any other mix or type of trauma; frequency of trauma exposure (i.e., single-event versus multiple or repeated trauma exposure); country status (as defined by the World Health Organization, i.e., low- or middle-income country vs high-income country); the gender of participants in each study (> 50% female vs other; 100% female vs other); and the age of the sample (mean ≥ 16.0 years vs < 16 years; this cut-off was selected so that we could examine whether removing the older samples, whose age range was more likely to go above 18 and may therefore have included some young adults, had any effect on the pattern of results obtained). The moderator analysis for single vs multiple/repeated trauma exposure was repeated but restricted to case–control studies to ensure a greater accuracy of trauma exposure characteristics. Other moderator analyses were conducted to consider whether methodological aspects of the included studies influenced the results: whether self-concept was measured using the Rosenberg Self-Esteem Scale (RSES, 1965), the most commonly used measure of self-concept (vs any other valid measure of self-concept); study design type (case–control studies, where there was a clear trauma-exposed group and a control or comparison group, vs cross-sectional studies); and study quality (high-quality vs low/medium-quality studies).

We were not able to conduct all our planned moderator analyses. Not all studies provided consistent data on socioeconomic status and age at trauma exposure. Additionally, there was considerable variation in how trauma exposure was measured between the studies. Due to these large discrepancies in moderator variables, we decided to not analyze socioeconomic status, age at trauma exposure, and measurement of trauma exposure.

### Publication Bias

Publication bias refers to the relationship between the choice to publish a paper and the results (Begg, 1994), which can lead to biased results. To evaluate publication bias, an inspection of the funnel plots and their statistical asymmetry tests were calculated. The funnel plots were used to graphically explore publication bias and the following tests were used to evaluate this statistically: Egger’s test of intercept (Egger et al., 1997) and the trim-and-fill procedure (Duval & Tweedie, 2000).

## Results

### Search Results

Overall, 4430 studies were identified through our searches and 134 met inclusion criteria (see Fig. 1). Reasons for inclusion and exclusion for full-text studies reviewed are provided in Fig. 1.

### Study Characteristics

Ninety-five studies were included in the final review which yielded a total of 134 independent effect sizes. The total sample size was 255,334 participants with sample sizes from individual studies ranging from 14 to 81,247 participants. Characteristics of the studies included in the meta-analysis (sample size, mean age, percent female, type of study, country, type of trauma exposure, single or repeated trauma, and measure of trauma exposure and measure of self-concept) are included in Supplementary Material 3. The mean age of the included studies (where mean age was reported and weighted by sample size) was 14.8.

### Meta-analysis of All Data

A random-effects meta-analysis of 134 independent effect sizes from 134 studies indicated a small effect size for the relationship between trauma exposure and self-concept, (r = − 0.20, 95% CI − 0.22, − 0.18). There was significant heterogeneity (Q = 3887.5, p < 0.001, *I*^2^ = 96%); the 95% prediction interval crossed the line of no effect (− 0.42, 0.05) suggesting that future studies could conceivably show no effect. Effect sizes for each individual study are reported in Supplementary Material 4.

### Moderator Analysis

There were enough studies (more than 10) to conduct moderator analysis. Table 1 shows the results of all the moderator analyses. This includes correlation coefficients, 95% confidence intervals, 95% prediction intervals, Cochran’s Q, *I*^2^ values, and the omnibus p-value for the significance of the moderation test (meta-regression). Four significant moderating effects were found for the relationship between trauma exposure and negative self-concept. A significantly stronger effect size was found for studies looking at only sexual abuse (r =− 0.24, k = 36) compared to any other trauma exposure type (r = − 0.18, k = 98). Forest plots for each of these sub-groups are presented in Supplementary Material 5 and 6. A significantly stronger effect size was found for studies looking at multiple or repeated trauma exposure (r = − 0.20, k = 129) compared to single-event trauma exposure (r = − 0.08, k = 5). When limiting to only case–control studies, this significantly stronger effect size for multiple or repeated trauma exposure (r = − 0.21, k = 45) compared to single-event trauma exposure [(r = − 0.10, k = 4) was maintained. No significant moderator effect was found for the relationship between trauma exposure and negative self-concept for study design (i.e., case–control vs cross-sectional), country status (i.e., high-income country vs low- and middle-income countries), gender, self-concept measure used, or mean age over 16 years old. A further analysis considered whether sample mean age (where reported; k = 95) moderated the relationship between self-concept and trauma exposure; this was not significant (p = 0.66). Additionally, there was no significant moderating effect between low- and medium-quality studies compared to high-quality studies.

**Table 1:** Results of regression analysis for relationship between trauma exposure and self-concept (including moderators)

	k	N	r	CI	PI	Q	I^2^	Moderator omnibus test p-value
*Overall*	134	255,334	− 0.20	− 0.22, − 0.18	− 0.42, 0.05	3887.5^***^	96%	
*Trauma type*								0.04^*^
*CSA*	36	47,049	− 0.24	− 0.28, − 0.20	− 0.44, − 0.02	1384.7^***^	93%	
*Mixed*	98	208,285	− 0.18	− 0.21, − 0.16	− 0.41, 0.06	1813.2^***^	97%	
*Measure type*								0.90
RSES	67	209,788	− 0.20	− 0.23, − 0.17	− 0.44, 0.07	3389.0^***^	98%	
Non-RSES	67	45,546	− 0.19	− 0.22, − 0.16	− 0.38, 0.01	417.1^***^	88%	
*Type of trauma*								0.05^*^
Single	5	8337	− 0.08	− 0.19, − 0.02	− 0.32, 0.16	57.7^***^	93%	
Multiple/repeated	129	246,997	− 0.20	− 0.22, − 0.18	− 0.42, 0.04	3803.1^***^	96%	
*Type of trauma (CC)*								0.04^*^
Single	4	7,940	− 0.10	− 0.24, 0.05	− 0.38, 0.21	54.9^***^	95% 79%	
Multiple/repeated	45	16,997	− 0.21	− 0.25, − 0.18	− 0.40, − 0.01	205.1^***^		
*Type of study*								0.68
Case–Control	49	24,937	− 0.20	− 0.24, − 0.17	− 0.40, 0.02	273.7^***^	86%	
Cross-sectional	85	230,397	− 0.19	− 0.22, − 0.17	− 0.43, 0.06	3605.7^***^	98%	
*Country status*								0.22
LMIC	36	58,274	− 0.22	− 0.26, − 0.18	− 0.45, 0.04	780.8^***^	96%	
High income	98	197,060	− 0.19	− 0.21, − 0.16	− 0.41, 0.05	3102.3^***^	96%	
*Gender*								0.96
50% female	85	152,876	− 0.20	− 0.22, − 0.17	− 0.40, 0.02	828.3^***^	94%	
Mix	42	88,688	− 0.20	− 0.24, − 0.16	− 0.45, 0.08	2582.3^***^	98%	
*Gender*								0.39
100% female	22	6892	− 0.22	− 0.27, − 0.16	− 0.41, − 0.01	88.9^***^	75%	
Mix	105	235,093	− 0.19	− 0.22, − 0.17	− 0.42, 0.06	3769.9^***^	97%	
*Age*								0.83
Up to 16 years	87	109,051	− 0.20	− 0.23, − 0.17	− 0.42, 0.05	2781.7^***^	95%	
16 years or greater	23	33,848	− 0.19	− 0.25, − 0.13	− 0.44, 0.08	399.9^***^	96%	
*Quality of studies*								0.29
High	76	209,264	− 0.21	− 0.24, − 0.18	− 0.44, 0.05	3422.0^***^	98%	
Medium or low	58	46,070	− 0.18	− 0.21, − 0.15	− 0.38, 0.04	355.5^***^	90%	

*Note* CC = case–control studies only; CI = confidence interval; CSA = childhood sexual abuse; k = number of effect sizes; LMIC: Low- and middle-income countries; N = number of participants; PI = prediction interval; r = Pearson’s r correlation coefficient, pooled estimate. ^*^p < 0.05; ^***^p < 0.001

All sub-groups were characterized by significant heterogeneity. Moreover, the 95% prediction intervals for each subgroup crossed the line of no effect, with the exception of the studies that focused on children exposed to sexual abuse, studies that used a case–control design comprised participants exposed to multiple or repeated trauma, and studies that entirely comprised female participants.

### Publication Bias

Publication bias analyses yielded a mixed picture. While visual inspection of the funnel plot was suggestive of asymmetry (aside from one outlier; see Supplementary Material 7), the Egger’s test of funnel plot asymmetry suggested significant asymmetry (z = -2.13, p = 0.033). The trim-and-fill procedure did not suggest that there were missing studies.

## Discussion

The current meta-analysis explored the relationship between trauma exposure and self-concept in children and adolescents. To the authors’ knowledge, this is the largest and only study to meta-analyze this relationship in children and adolescents. Results from pooling 134 independent effect sizes from the same number of studies found a significant negative effect of the relationship between trauma exposure and self-concept; this size of the effect was found to be small (r =  − 0.20) and in the expected direction, i.e., greater trauma exposure was associated with poorer self-concept.

Further analyses were undertaken to understand what study-level characteristics may be moderating this relationship. Analysis of moderators found that two study-level factors moderated this relationship: trauma exposure frequency (single vs repeated) and sexual abuse (vs other types of abuse). The size of the effect was larger for studies that looked at multiple and repeated trauma exposure compared to single-event trauma exposure (e.g., earthquakes); this effect remained even when limiting study designs to case–control studies. This is in line with findings in the adult literature that indicate that increased exposure to trauma is related to poorer outcomes (Sowder et al., 2018). However, it is important to note that there were only five single incident trauma exposure studies in the review.

It is also noteworthy that the relationship between trauma exposure and self-concept was moderated by studies that only looked at child sexual abuse compared to any other mix of trauma exposure. These results suggest that it is not only the amount of exposure to the trauma, but the nature of the trauma exposure that has a relationship with one’s self-concept; this is supported by findings in the adult literature that state that child sexual abuse is linked to worse outcomes later in life, relative to other trauma exposure types (Maniglio, 2009).

That no other study-level characteristics moderated the relationship between trauma exposure and negative self-concept also warrants comment. While females are more likely to be exposed to trauma and maltreatment (Gallo et al., 2018; Gwadz et al., 2007; Halpern et al., 2018) and previous research has found that females are more likely to experience negative outcomes following trauma exposure when compared to males (Holbrook et al., 2002), in the current meta-analysis, gender (whether 100% female or 50% female compared to a mix) did not moderate the relationship. This suggests that girls are no more likely than boys to have a worse impact on self-concept after exposure to trauma or vice versa. The relationship between trauma exposure and self-concept was found to be present in both low- and middle-income countries (LMICs) and high-income countries, stressing the global importance of this mechanism.

Finally, another important finding was that, while the Rosenberg Self-Esteem Scale (1965) has been most commonly used to measure self-concept, there was no moderating effect between using this measure compared to any other measure of self-concept. A final finding was that study quality did not affect the pattern of results; the difference between high versus low- and medium-quality studies did not change the size of the effect. This is an important finding as it suggests that regardless of study quality, the relationship between trauma exposure and negative self-concept is robust.

It is also important to note the issue of publication bias in the current sample. The two tests for publication bias show differing results which suggests there may be a possibility of publication bias affecting the results, particularly with a lack of studies in the positive direction. However, given the large sample size and the number of large studies in the analysis, publication bias is unlikely to have a large effect on the results. Moreover, given the significant heterogeneity in the outcomes, the tests of asymmetry may not be the most appropriate way to interpret publication bias (Ioannidis & Trikalinos, 2007).

It is important to highlight the direction and scale of the effect. As the current review looked at the relationship between exposure to traumatic events and negative self-concept using correlations, it is not possible to show causation, e.g., that trauma exposure causes negative self-concept. While it may be plausible to suggest that increased trauma exposure may lead to negative self-concept, it is also possible that children and adolescents with lower self-concept may be more vulnerable to trauma exposure. The size of the relationship is another interesting finding; the magnitude of the relationship between trauma exposure and negative self-concept is relatively small. While this could be explained by the large number of studies included in the analysis and the large variation between them, this could also suggest that trauma exposure and maltreatment may not have as detrimental of a relationship to one’s sense of self as one may expect. Nasvytiene et al. (2012) found that resilience could play a role here; in their meta-analysis, they found that individual characteristics, such as positive self-esteem, contributed to resilience following maltreatment. It is also important to consider how the effect of this relationship may be dispersed. It is possible that for many children and adolescents there is no relationship between trauma exposure and negative self-concept. However, some children may get a larger impact and have a greater relationship between mental health difficulties and trauma exposure, consistent with some findings in adolescents (e.g., Salami, 2010) and the broader literature showing a strong relationship between self-related appraisals and PTSD severity in trauma-exposed adults (Gomez de la Cuesta et al., 2019). The current meta-analysis, however, does not capture this information and is not able to determine these nuances of the relationship between trauma exposure and negative self-concept.

## Limitations

The present study had some important limitations. It should be noted that the current findings found a large degree of heterogeneity in the main meta-analysis and moderator analyses. There are many factors which have likely contributed to this large heterogeneity: differing methodological procedures used, large differences in the number and experiences of participants in each study, and different measures used. Moderator analysis was performed to understand the source of the heterogeneity; however, this did not completely resolve this issue and therefore, it is still unclear what the precise causes for the heterogeneity is. The type of trauma exposure measure could have contributed to this but it was not possible to explore this in the current analysis.

A further limitation is the large degree of missing information in some of the studies pertaining to study characteristics; many studies did not report age at trauma exposure or details on sociodemographic factors which meant not all of the prospective moderators identified could be evaluated. Additionally, the coding of studies for the purpose of the moderator analyses as ‘repeated or multiple’ in terms of nature of trauma exposure is a potential limitation. In particular, there were inconsistencies among the studies in the reporting of trauma exposure. For example, some studies reported trauma as a continuous variable where the higher the number, the more exposure to trauma. In these cases, it was not possible to pick apart those individuals who had scores of “0” indicating no trauma exposure within the study and therefore the study as a whole was coded as “multiple or repeated trauma exposure.” To account for these inconsistencies a further moderator analysis was conducted limiting the analysis to only case–control studies.

### Clinical Implications and Future Research

Despite these limitations, the current meta-analysis has important implications for practice. The significant but small effect found between trauma exposure and negative self-concept in children and adolescents provides an understanding into the potential role that self-concept may play. Given the significant main effect and the finding that the nature, exposure, or frequency of trauma can moderate this relationship, early identification of those who are at an increased risk of exposure to trauma and maltreatment is important. Given the mediating role that self-concept has on future psychopathology (Ehlers & Clark, 2000; Gomez de la Cuesta et al., 2019; Salami, 2010), it is imperative to understand the role that being exposed to trauma may have on the self-concept of children and adolescents.

These findings additionally highlight how self-concept may be an important mechanism to consider after exposure to a traumatic event(s), especially considering its mediating relationship and link to further mental health difficulties (Evans et al., 2015). This highlights a need to provide tools and interventions to directly target negative self-concept as a means to potentially help with other post-trauma reactions. These can include resources such as access to psychological therapies, such as Trauma-Focused Cognitive-Behavioral Therapy (Cohen et al., 2016), and psychoeducational resources, as well as more cost-effective tools to take into consideration both cultural factors and the nature of the trauma exposure.

The current meta-analysis highlighted a lack of studies measuring single-event trauma exposure. To gain a more in depth and accurate understanding of the effect of self-concept with single-event trauma exposure, more studies are needed in children and adolescents to evaluate this relationship. Given the scope of the current review, studies were not restricted based on age at trauma exposure. A further meta-analysis looking at trauma exposure and self-concept with a focus on age at trauma exposure may help reveal further nuances of this relationship.

The finding that sexual abuse in childhood and multiple/repeated trauma yielded a statistically strong effect is broadly consistent with ICD-11’s (WHO, 2018) definition of trauma exposure for the diagnosis of Complex PTSD. However, these stronger effects were not very large which suggests that it may be that a broader range of trauma exposure, not just trauma exposure that is severe and recurrent, that has a significant impact on self-concept. This gives important clinical implications for the importance of focusing on the impact of the trauma exposure for the individual rather than just those who experienced more severe and recurrent forms of trauma exposure.

## Conclusion

In summary, the current meta-analysis found a significant relationship between trauma exposure and self-concept in children and adolescents under 18 years old, where increased trauma exposure was related to negative or lower self-concept. This relationship was moderated by type of trauma exposure (sexual abuse or any mixed trauma) and whether this was single-event trauma exposure or a multiple and repeated trauma exposure. However, there was heterogeneity in the results with a mixed picture of publication bias. Overall, while it is not plausible to suggest causation from these results, the findings highlight a need to focus on all types of trauma exposure and provide resources and interventions to help improve the self-concept of those exposed or at risk to trauma exposure and maltreatment.

### Supplementary Information

Below is the link to the electronic supplementary material.

Supplementary file1 (DOCX 1312 kb)
