# Supplementary material for: The Relationship Between Negative Self-Concept, Trauma, and Maltreatment in Children and Adolescents: A Meta-Analysis
Source: Clin Child Fam Psychol Rev. 2024 Feb 22;27(1):220–34. doi: 10.1007/s10567-024-00472-9 (PMC10920440; doi:10.1007/s10567-024-00472-9)

*Supplementary Material 1. Quality checklist (Adapted Strobe Statement Quality Checklist)*

| Population 1: Was the study population, participants and setting well defined? | 0 = sample characteristics, trauma and self-concept variables not reported in any detail  1= sample characteristics, trauma and self-concept variable are reported with limited detail  2= sample characteristics, trauma and self-concept variable are described in detail (including type of study, country of origin, age, gender, ethnicity) |
| --- | --- |
| Population 2: Was the sampling carried out appropriate to the study design, where likelihood of sampling bias minimised? | 0 = convenience sampling  1= admissions to a pediatric unit/hospital admission, etc.  2= random sampling of those exposed to traumatic event and those not or random sampling of population |
| Methods 1: Was there a validated measure of trauma or was the trauma group appropriately recruited? | 0 = not reported  1= yes or no questions to trauma by researchers/not valid measure of trauma  2= trauma appropriately recruited/valid measure of trauma |
| Methods 2: Was the measure of self-concept a valid and reliable measure? | 0 = not reported  1= any other self-concept measure  2=RSES or adapted RSES |
| Methods 3: Was the likelihood of non-response bias minimised? | 0 = not reported or unclear  1= response rate around 20% or minimal explanation of how this was treated  2= response rate at least 40%, or analysis performed to show no significant difference between responders and non-responders |
| Analysis 1: Was the sample size adequate? | 0 = justification of sample size not reported or very small sample size  1= sample size adequate but did not refer to sample size calculations or consider previous studies/minimal information on sample size, 2= sample size adequate and based on sample size/power calculations or reference to other studies |
| Analysis 2: Was there appropriate statistical analysis used? | 0=statistical methods were inappropriate or the study lacked information on the statistical methodology when reporting the data or size of effect  1= statistical method appropriate but with some missing information e.g. confidence intervals  2= statistical analysis were appropriate, confidence intervals reported with 95% bias or equivalent for statistical analysis |
| Overall Risk of Bias | /14 |

*Supplementary Material 2. Quality ratings.*

| **Author (Year)** | **Q1** | **Q2** | **Q3** | **Q4** | **Q5** | **Q6** | **Q7** | **Total (0-14)** | **Quality Rating** |
| --- | --- | --- | --- | --- | --- | --- | --- | --- | --- |
| Ackard (2002) | 2 | 2 | 1 | 2 | 2 | 1 | 2 | 12 | High |
| Aloba (2020) | 2 | 0 | 2 | 2 | 2 | 1 | 2 | 11 | High |
| Arslan (2016) | 1 | 2 | 2 | 2 | 0 | 1 | 1 | 9 | High |
| Asgeirsdottir (2010) | 1 | 0 | 1 | 2 | 2 | 1 | 1 | 8 | Moderate |
| Ayhan (2023) | 1 | 2 | 2 | 1 | 2 | 1 | 2 | 11 | High |
| Baeg (2020) | 2 | 2 | 2 | 2 | 0 | 1 | 2 | 11 | High |
| Bagley (1992) | 1 | 2 | 1 | 1 | 0 | 1 | 1 | 7 | Moderate |
| Bailey (2005) | 2 | 2 | 2 | 2 | 0 | 1 | 1 | 10 | High |
| Bernard-Bonnin (2008) | 1 | 1 | 2 | 1 | 0 | 1 | 1 | 7 | Moderate |
| Bolger (1998) | 1 | 0 | 2 | 1 | 0 | 0 | 1 | 5 | Moderate |
| Brown (2019) | 2 | 2 | 2 | 2 | 0 | 1 | 2 | 11 | High |
| Burack (2006) | 2 | 0 | 2 | 1 | 0 | 1 | 0 | 6 | Moderate |
| Cecil (2001) | 1 | 1 | 2 | 2 | 0 | 1 | 2 | 9 | High |
| Cederbaum (2020) | 2 | 2 | 2 | 1 | 2 | 1 | 1 | 11 | High |
| Chang (2012) | 1 | 0 | 1 | 1 | 0 | 0 | 0 | 3 | Low |
| Chen (2019) | 2 | 2 | 2 | 2 | 0 | 1 | 2 | 11 | High |
| Chen-2022a | 1 | 2 | 2 | 2 | 2 | 1 | 2 | 12 | High |
| Chen-2022b | 1 | 2 | 1 | 2 | 2 | 1 | 1 | 10 | High |
| Choi (2016) | 2 | 1 | 2 | 2 | 0 | 1 | 2 | 10 | High |
| Daniel (2016) | 2 | 2 | 2 | 2 | 0 | 1 | 2 | 11 | High |
| Deb (2016) | 2 | 2 | 1 | 1 | 0 | 1 | 1 | 8 | Moderate |
| Dion (2021) | 2 | 2 | 1 | 1 | 0 | 1 | 2 | 9 | Moderate |
| Dion (2022) | 2 | 2 | 1 | 1 | 2 | 1 | 2 | 11 | High |
| Doku (2023) | 2 | 2 | 2 | 2 | 0 | 2 | 2 | 12 | High |
| Egan (1998) | 1 | 2 | 1 | 1 | 0 | 1 | 2 | 8 | Moderate |
| Elliott (1990) | 2 | 1 | 2 | 1 | 0 | 0 | 1 | 7 | Moderate |
| Esparza (1996) | 2 | 1 | 2 | 1 | 0 | 1 | 1 | 8 | Moderate |
| Esparza-Del Villar (2022) | 1 | 0 | 2 | 2 | 0 | 1 | 2 | 8 | Moderate |
| Flynn (2014) | 2 | 2 | 2 | 1 | 0 | 1 | 2 | 10 | High |
| Folayan (2020) | 1 | 2 | 2 | 2 | 0 | 2 | 1 | 10 | High |
| Fonseca de Freitas (2022) | 2 | 2 | 2 | 2 | 2 | 1 | 2 | 13 | High |
| Fu (2022) | 1 | 2 | 2 | 2 | 0 | 1 | 2 | 10 | High |
| Garduno (2022) | 2 | 1 | 1 | 1 | 0 | 1 | 2 | 8 | Moderate |
| Gauthier-Duchesne (2022) | 2 | 2 | 1 | 2 | 2 | 1 | 2 | 12 | High |
| Genç (2018) | 2 | 2 | 1 | 1 | 0 | 1 | 2 | 9 | High |
| German (1990) | 1 | 2 | 1 | 1 | 0 | 1 | 1 | 7 | Moderate |
| Gesinde (2011) | 1 | 2 | 1 | 1 | 0 | 1 | 2 | 8 | Moderate |
| Gewirtz-Meydan (2020) | 1 | 2 | 2 | 1 | 0 | 1 | 2 | 9 | High |
| Grayston (1992) | 1 | 1 | 2 | 1 | 0 | 1 | 1 | 7 | Moderate |
| Greger (2016) | 2 | 2 | 2 | 1 | 0 | 1 | 2 | 10 | High |
| Greger (2017) | 1 | 1 | 2 | 1 | 2 | 1 | 2 | 10 | High |
| Gunnlaugsson (2013) | 2 | 2 | 1 | 2 | 0 | 1 | 2 | 10 | High |
| Haj-Yahia (2002) | 2 | 2 | 2 | 2 | 0 | 1 | 2 | 11 | High |
| Herd (2022) | 2 | 1 | 2 | 1 | 0 | 1 | 2 | 9 | Moderate |
| Hibbard (1988) | 1 | 2 | 1 | 2 | 0 | 1 | 1 | 8 | Moderate |
| Hibbard (1992) | 2 | 1 | 2 | 1 | 0 | 1 | 1 | 8 | Moderate |
| Jankowiak (2021) | 1 | 2 | 1 | 2 | 2 | 2 | 1 | 11 | High |
| Jezl (1996) | 2 | 2 | 2 | 2 | 0 | 1 | 1 | 10 | High |
| Johnson-2001 | 1 | 1 | 2 | 1 | 0 | 1 | 1 | 7 | Moderate |
| Jonsson (2019) | 2 | 2 | 1 | 2 | 2 | 1 | 2 | 12 | High |
| Ju (2018) | 1 | 2 | 1 | 1 | 0 | 1 | 2 | 8 | Moderate |
| Kaufman (1989) | 1 | 1 | 2 | 1 | 0 | 2 | 1 | 8 | Moderate |
| Kim (2017) | 2 | 2 | 2 | 1 | 1 | 1 | 1 | 10 | High |
| Kim (2004) | 1 | 2 | 2 | 1 | 0 | 1 | 2 | 9 | High |
| Kim (2006) | 2 | 2 | 2 | 1 | 0 | 2 | 1 | 10 | High |
| Kim (2020) | 2 | 2 | 1 | 1 | 0 | 1 | 1 | 8 | Moderate |
| Kim (2021) | 2 | 2 | 1 | 2 | 0 | 1 | 2 | 10 | Moderate |
| Kocturk (2017) | 1 | 2 | 1 | 1 | 0 | 1 | 2 | 8 | Moderate |
| Lam (2015) | 1 | 0 | 1 | 1 | 0 | 1 | 2 | 6 | Moderate |
| Lau (2003) | 1 | 2 | 1 | 1 | 0 | 1 | 2 | 8 | Moderate |
| Lee (2021) | 2 | 0 | 2 | 2 | 0 | 1 | 2 | 9 | Moderte |
| Leeson (2011) | 2 | 1 | 2 | 1 | 0 | 1 | 1 | 8 | Moderate |
| Li (2009) | 2 | 2 | 2 | 2 | 0 | 1 | 1 | 10 | High |
| Li (2023) | 2 | 2 | 2 | 2 | 2 | 1 | 2 | 13 | High |
| Lim (2017) | 1 | 2 | 1 | 2 | 0 | 1 | 2 | 9 | High |
| Lim (2020) | 2 | 2 | 2 | 2 | 0 | 1 | 2 | 11 | High |
| Lin (2011) | 2 | 2 | 2 | 2 | 0 | 1 | 2 | 11 | High |
| Liu (2023) | 2 | 2 | 2 | 2 | 2 | 1 | 2 | 13 | High |
| Luo (2020) | 1 | 2 | 2 | 2 | 0 | 1 | 2 | 10 | High |
| Lynch (1998) | 1 | 2 | 2 | 1 | 0 | 1 | 2 | 9 | High |
| Ma (2014) | 2 | 2 | 2 | 1 | 0 | 1 | 2 | 10 | High |
| Malik (2016) | 1 | 2 | 1 | 2 | 0 | 1 | 1 | 8 | Moderate |
| Mannarino (1989) | 1 | 2 | 2 | 1 | 0 | 1 | 1 | 8 | Moderate |
| Maruyama (2022) | 2 | 2 | 2 | 2 | 2 | 1 | 1 | 12 | High |
| Maskell (2013) | 2 | 1 | 2 | 1 | 0 | 1 | 1 | 8 | Moderate |
| Matejcek (1983) | 1 | 1 | 1 | 1 | 0 | 1 | 1 | 6 | Moderate |
| Medora (1993) | 2 | 2 | 1 | 1 | 0 | 1 | 1 | 8 | Moderate |
| Mennen (1994)a | 2 | 1 | 2 | 1 | 0 | 1 | 1 | 8 | Moderate |
| Mennen (1994)b | 2 | 1 | 2 | 1 | 0 | 1 | 1 | 8 | Moderate |
| Mennen (1993) | 2 | 1 | 2 | 1 | 0 | 1 | 1 | 8 | Moderate |
| Moyer (1997) | 1 | 2 | 2 | 1 | 0 | 1 | 2 | 9 | High |
| Mwakanyamale (2019) | 1 | 2 | 2 | 2 | 2 | 2 | 2 | 13 | High |
| Nguyen (2019) | 2 | 2 | 1 | 2 | 0 | 1 | 2 | 10 | High |
| Nguyen (2010) | 2 | 0 | 2 | 2 | 0 | 1 | 2 | 9 | High |
| Oates (1994) | 1 | 2 | 2 | 1 | 0 | 1 | 1 | 8 | Moderate |
| Oates (1985) | 1 | 2 | 2 | 1 | 0 | 0 | 0 | 6 | Moderate |
| O'Keefe (1998) | 1 | 2 | 1 | 2 | 0 | 1 | 1 | 8 | Moderate |
| Orr (1985) | 1 | 1 | 2 | 1 | 0 | 1 | 1 | 7 | Moderate |
| Pantelewicz (2021) | 2 | 1 | 1 | 0 | 0 | 1 | 1 | 6 | Moderate |
| Parent (2022) | 2 | 0 | 2 | 1 | 0 | 1 | 2 | 8 | Moderate |
| Park (2018) | 2 | 2 | 2 | 2 | 0 | 1 | 1 | 10 | High |
| Rana (2020) | 2 | 2 | 2 | 2 | 2 | 2 | 2 | 14 | High |
| Reid-Russell (2022) | 2 | 2 | 2 | 1 | 0 | 1 | 2 | 10 | High |
| Reyes (2008) | 2 | 2 | 2 | 1 | 0 | 2 | 2 | 11 | High |
| Ronzon-Tirado (2022) | 2 | 2 | 1 | 2 | 0 | 1 | 1 | 9 | Moderate |
| Rust (1991) | 1 | 1 | 2 | 1 | 0 | 1 | 1 | 7 | Moderate |
| Ryu (2023) | 1 | 2 | 1 | 2 | 0 | 1 | 2 | 9 | Moderate |
| Saigh (2008) | 2 | 1 | 2 | 1 | 0 | 1 | 1 | 8 | Moderate |
| Salazar (2004) | 2 | 2 | 2 | 2 | 0 | 1 | 1 | 10 | High |
| Sayar (2005) | 1 | 2 | 1 | 2 | 0 | 1 | 1 | 8 | Moderate |
| Scheer (2022) | 2 | 1 | 1 | 2 | 2 | 2 | 2 | 12 | High |
| Schlechter (2021) | 2 | 2 | 1 | 2 | 0 | 1 | 2 | 10 | High |
| Sciacca (2023) | 1 | 2 | 1 | 2 | 0 | 1 | 2 | 9 | Moderate |
| Sevenoaks (2022) | 1 | 1 | 2 | 1 | 0 | 1 | 1 | 7 | Moderate |
| Shah (2021) | 2 | 2 | 2 | 2 | 0 | 2 | 2 | 12 | High |
| Shattnawi (2022) | 1 | 0 | 2 | 2 | 2 | 2 | 1 | 10 | High |
| Shen (2015) | 1 | 2 | 2 | 2 | 0 | 1 | 2 | 10 | High |
| Skeen (2016) | 2 | 2 | 2 | 2 | 0 | 1 | 2 | 11 | High |
| Smith (2018) | 1 | 2 | 1 | 1 | 0 | 1 | 2 | 8 | Moderate |
| Soler (2012) | 2 | 2 | 2 | 2 | 0 | 1 | 2 | 11 | High |
| Stern (1995) | 2 | 1 | 2 | 1 | 0 | 1 | 1 | 8 | Moderate |
| Sturkie (1987) | 1 | 2 | 2 | 1 | 0 | 1 | 1 | 8 | Moderate |
| Suzuki (2015) | 1 | 1 | 2 | 2 | 0 | 1 | 1 | 8 | Moderate |
| Swanston (1997) | 2 | 1 | 2 | 1 | 0 | 1 | 2 | 9 | High |
| Tocker (2017) | 1 | 2 | 2 | 2 | 0 | 1 | 1 | 9 | High |
| Tong (1987) | 1 | 1 | 2 | 1 | 0 | 1 | 1 | 7 | Moderate |
| Toth (1992) | 1 | 0 | 2 | 1 | 0 | 0 | 1 | 5 | Moderate |
| Trickett (2011) | 2 | 2 | 2 | 1 | 0 | 1 | 1 | 9 | High |
| Turner (2010) | 1 | 2 | 1 | 2 | 0 | 1 | 2 | 9 | High |
| Vigil (2008) | 1 | 2 | 2 | 2 | 0 | 1 | 1 | 9 | High |
| Wang (2020) | 1 | 2 | 2 | 2 | 0 | 1 | 2 | 10 | High |
| Weiler (2019) | 2 | 2 | 2 | 1 | 0 | 1 | 2 | 10 | High |
| Wodarski (1990) | 2 | 2 | 2 | 1 | 0 | 1 | 2 | 10 | High |
| Wonderlich (2001) | 1 | 1 | 2 | 1 | 0 | 1 | 2 | 8 | Moderate |
| Wondie (2011) | 2 | 2 | 2 | 2 | 0 | 1 | 2 | 11 | High |
| Wu (2020) | 1 | 0 | 2 | 2 | 0 | 1 | 1 | 7 | Moderate |
| Wu (2023) | 1 | 2 | 2 | 2 | 2 | 0 | 1 | 10 | High |
| Yoder (2005) | 2 | 2 | 2 | 2 | 0 | 2 | 1 | 11 | High |
| Yoo (2021) | 1 | 2 | 1 | 2 | 0 | 0 | 1 | 7 | Moderate |
| Yu (2021) | 2 | 0 | 2 | 2 | 2 | 2 | 2 | 12 | High |
| Zeller (2015) | 2 | 2 | 2 | 1 | 0 | 1 | 2 | 10 | High |
| Zhang (2022) | 1 | 2 | 2 | 2 | 2 | 0 | 2 | 11 | High |
| Zhou (2019) | 1 | 2 | 2 | 2 | 0 | 1 | 2 | 10 | High |
| Zhu (2020) | 2 | 2 | 2 | 2 | 2 | 1 | 2 | 13 | High |

*Supplementary Table 3. Study Characteristics of all studies included in the meta-analysis*

| **First Author and Year** | **N** | **Mean Age or Age Range** | **Female (%)** | **Type of Study** | **Country** | **Type of Trauma** | **Single or Multiple/ repeated Trauma** | **Measure of Trauma Exposure** | **Measure of Self-concept** | |
| --- | --- | --- | --- | --- | --- | --- | --- | --- | --- | --- |
| Ackard (2002) | 81247 | 9^th^ & 12^th^ Grade | 50% | CS | USA | Date Related Violence | M/R | Records | Adapted RSES | |
| Aloba (2020) | 1337 | 15.2 | 55% | CS | Nigeria | PA, EA, CSA, PN, EN | M/R | Questionnaire | RSES | |
| Arslan (2016) | 1352 | 16.5 | 54% | CS | Turkey | PsM | M/R | Questionnaire | RSES | |
| Asgeirsdottir (2010) | 9113 | 17.2 | 51% | CS | Iceland | CSA | M/R | Questionnaire | RSES | |
| Ayhan (2023) | 270 | 10.7 | 52% | CS | Turkey | N | M/R | Questionnaire | CSEI | |
| Baeg (2020) | 605 | 7^th^ to 9^th^ grade | 49% | L | South Korea | Peer Victimization | M/R | Questionnaire | RSES | |
| Bagley (1992) | 369 | 14 to 16 | N/A | CS | Canada | Abuse at Home | M/R | Questionnaire | CSEI | |
| Bailey (2005) | 43 trauma, 107 control | 14.6 | 100% | L | USA | CSA | M/R | Questionnaire | RSES | |
| Bernard-Bonnin (2008) | 67 trauma, 67 control | 9.0, 9.3 | 100% | CC | Canada | CSA | M/R | Records | SPPC | |
| Bolger (1998) | 107 trauma, 107 control | 8 to 10 | 48% | L | USA | PA, CSA, EM, N | M/R | Records | SPPC | |
| Brown (2019) | 3070 trauma, 2796 control | 14.3 & 14.8 | 48% | CC | Canada | Wildfire | Single | Records | RSES | |
| Burack (2006) | 49 trauma, 49 control | 10.3, 15 | 23% | CC | Canada | PA, N (lack of supervision), CSA, PN, exposure to violence | M/R | Records | SPPA & SPPC | |
| Cecil (2001) | 249 | 16.5 | 100% | CS | USA | CSA | M/R | Questionnaire | RSES | |
| Cederbaum (2020) | 454 | 9 to 13 | 47% | L | USA | CM | M/R | Records | SPPA | |
| Chang (2012) | 14 | 12.4 | 100% | CS | Nicaragua | PA | M/R | Questionnaire | Questionnaire | |
| Chen (2019) | 580 | 11.7 | 49% | CS | China | EA | M/R | Questionnaire | RSES | |
| Chen (2022)a | 417 | 16.8 | 48% | CS | China | PA, N | M/R | Questionnaire |  | |
| Chen (2022)b | 941 | 12 to 14 | 40% | L | China | Mixed | M/R | ACE | RSES | |
| Choi (2016) | 92 trauma, 351 control | 5 to 13 | 77 %& 52% | CC | Korea | CSA | M/R | Records | RSES | |
| Daniel (2016) | 259 trauma, 281 control | 16.2, 16.1 | 57% & 28.% | L | Israel | Terrorist attack | Single | Recruitment | RSES | |
| Deb (2016) | 370 | 16.7 | 51% | CS | India | Violence (psychological, physical, sexual), multiple abuse | M/R | Records | SCS | |
| Dion (2021) | 227 | 15.6 | 56% | CS | Canada | Dating violence | M/R | Questionnaire | Questionnaire | |
| Dion (2022) | 1802 | 14.7 | 42% | CS | Canada | SA, EA, PA, N, domestic violence | M/R | Questionnaire | Questionnaire | |
| Doku (2023) | 291 | 13 | 51% | CS | Ghana | CM | M/R | CTS-C | RSES | |
| Egan (1998) | 189 | 3rd to 7th grade | 51% | CS | USA | Peer Victimization | M/R | Questionnaire | PHSCS | |
| Elliott (1990) | 17 trauma, 17 control | 6 to 13 | 71% & 65% | CC | USA | CSA | M/R | Records | PHSCS | |
| Esparza (1996) | 54 trauma, 69 control | 13 to 20 | 100% | CC | USA | CSA | M/R | Questionnaire | Questionnaire | |
| Esparza-Del Villar (2022) | 526 | 16.5 | 49% | CS | Mexico | V, CA, N, partner violence | M/R | Questionnaire | RSES | |
| Flynn (2014) | 635 | 13 to 15 | 41% | L | USA | CSA, PA, EM, N | M/R | Records | SPPA | |
| Folayan (2020) | 1001 | 13.4 | 55% | CS | Nigeria | Mixed | M/R | ACE | RSES | |
| Fonesca de Freitas (2022) | 2975 | 16.6 | 54% | CS | Portugal | Physical, verbal, social Victimization, attacks of property | M/R | Questionnaire | RSES | |
| Fu (2022) | 4313 | 10.9 | 46% | L | China | Family M, peer victimization | M/R | CTQ-SF & MPVS | RSES | |
| Garduno (2022) | 555 | 14.96 | 51% | CS | USA | Mixed | M/R | Questionnaire | Questionnaire | |
| Gauthier-Duchesne (2022) | 8194 | 14 to 18 | 57.8% | CS | Canada | CSA | M/R | Questionnaire | SDQ | |
| Genç (2018) | 3193 | Grades 7-12 | 55% | L | USA | CM | M/R | Questionnaire | Questionnaire | |
| German (1990) | 40 | 14.4 | 100% | CC | USA | CSA | M/R | Records | PHSCS | |
| Gesinde (2011) | 480 | NR | 53% | CS | Nigeria | EM | M/R | Questionnaire | Questionnaire | |
| Gewirtz-Meydan (2020) | 828 | 15.5 | 38% | L | USA | CSA | M/R | Questionnaire | RSES & Questionnaire | |
| Grayston (1992) | 34 trauma, 35 control | 7 to 12 | 100% | CC | Canada | CSA | M/R | Records | SEI | |
| Greger (2016) | 237 trauma, 1017 control | 17, 14.1 | 65%, 45% | CC | Norway | Witness Violence, Family & Community Violence | M/R | Questionnaire | Questionnaire | |
| Greger (2017) | 400 | 16.8 | 58% | CS | Norway | CM, CSA, Witness Violence, Family & Violence | M/R | CAPA | SPPA | |
| Gunnlaughsson (2013) | 3515 | 14 to 15 | 49% | CS | Iceland | Physical violence at home | M/R | Questionnaire | RSES | |
| Haj-Yahia (2002) | 1640 | 17.2 | 52% | CS | Israel | PA | M/R | Questionnaire | RSES | |
| Herd (2022) | 498 | 15.3 | 100% | CS | USA | CSA, PA, N, other trauma | M/R | Records & CTI | SPPA | |
| Hibbard (1988) | 712 | 13.5 | 50% | CS | USA | CSA, PA | M/R | Questionnaire | RSES | |
| Hibbard (1992) | 82 | 14.5 | 50% & 46% | L | USA | N, CSA, PA, EA, | M/R | Records | Questionnaire | |
| Jankowiak (2021) | 1451 | 13 to 16 | 61% | CS | Mixed | PA, SA, dating violence | M.R | Questionnaire | RSES | |
| Jezl (1996) | 257 | 9^th^ and 12^th^ grade | 50% & 51% | CS | USA | PsM, PA | M/R | Questionnaire | RSES | |
| Johnson (2001) | 60 trauma, 60 control | 17 | 100% | CC | USA | CSA | M/R | Recruitment | SEI | |
| Jonsson (2019) | 5715 | 18 | 55% | CS | Sweden | CSA, EA, PA | M/R | Records | RSES | |
| Ju (2018) | 2844 | 4^th^ grade | NR | L | Korea | PhM | M/R | Records | Questionnaire | |
| Kaufman (1989) | 70 trauma, 67 control | 5 to 11 | NR | CC | USA | N, EA, PA | Multiple/Repeated | Questionnaire | Questionnaire | |
| Kim (2017) | 802 | 14.1 | 35% | CS | South Korea | PA, EA, CSA, | M/R | Questionnaire | RSES | |
| Kim (2004) | 206 trauma, 139 control | 9.2 | 36% | CC | USA | CSA, PA, PN, EM | M/R | Records | CSEI | |
| Kim (2006) | 251 | 8.5 | 36% | L | USA | EM, PN, PA, CSA | M/R | Records | CSEI | |
| Kim (2020) | 2844 | 9 | 48% | L | South Korea | Peer Victimisation | M/R |  |  | |
| Kim (2021) | 4328 | 11 | 48% | CS | South Korea | School Violence | M/R |  |  | |
| Kocturk (2017) | 210 | 15.9 | 100% | CC | Turkey | CSA, sexual revictimisation | M/R | forensic interviews | Questionnaire | |
| Lam (2015) | 980 | 14.8 | 51% | CS | Hong Kong | CSA | M/R | Questionnaire | CF-SEI | |
| Lau (2003) | 489 | 13 to 15 | 38% | CS | Hong Kong | PM | M/R | Questionnaire | SPPA | |
| Lee (2021) | 2321 | 15.9 | 39% | CS | Taiwan | Victimisation | M/R | Questionnaire | RSES | |
| Leeson (2011) | 50 | 11.2 | 54% | CC | Australia | CM | M/R | Questionnaire | CSEI | |
| Li (2009) | 1625 | 12.9 | 49% | CS | China | 21 different types of trauma (HIV-related) | M/R | Questionnaire | RSES | |
| Li (2023) | 1957 | 13* | 49% | CS | China | CM | M/R | CTQ-SF | RSES | |
| Lim (2017) | 2351 | middle school students | 48% | L | Korea | PA, N | M/R | Questionnaire | RSES | |
| Lim (2020) | 1831 | 10.98 | 49% | L | South Korea | CM | M/R | Questionnaire | RSES | |
| Lin (2011) | 683 | 12.9 | 52% | CS | China | CSA | M/R | Questionnaire | RSES | |
| Liu (2023) | 1064 | 9 to 14 | 50% | CS | China | Peer victimization | M/R | Questionnaire | RSES | |
| Luo (2020) | 1302 | 11.3 | 50% | CS | China | PsM | M/R | Questionnaire | RSES | |
| Lynch (1998) | 188 trauma, 134 control | 8.7, 8.8 | 43% & 34% | L | USA | CSA, PA, PN, EM | M/R | Questionnaire | CSEI | |
| Ma (2014) | 165 trauma, 201 control | 9 to 15 | 54%, 51% & 60% | CC | Hong Kong | CSA, PA, abuse | M/R | Records | CF-SEI | |
| Malik (2016) | 400 | 16.1 | 50% | CS | India | EM | M/R | Questionnaire | RSES | |
| Mannarino (1989) | 94 trauma, 75 control | 6 to 12 | 100% | CC | USA | CSA | M/R | Records | PHSCS | |
| Maruyama (2022) | 1949 | 15.7 | 48% | L | Brazil | Abuse at home | M/R | Questionnaire | RSES | |
| Maskell (2013) | 66 trauma, 1387 control | 12.7 | 75% | CC | Australia, New Zealand, USA | burn injury | Single | Records | PHSCS | |
| Matejcek (1983) | 228 | 12.2 | 49% | CS | Czech Republic | parental neglect & rejection aggression | M/R | Questionnaire | Questionnaire | |
| Medora (1993) | 121 | 12 to 19 | 100% | CS | USA | CSA | M/R | Records | Questionnaire | |
| Mennen (1994a) | 117 trauma, 1774 standardization | 13.8, 13.1 | 100% | CC | USA | CSA | M/R | Records | SPPC | |
| Mennen (1994b) | 75 trauma, 1174 standardization | 12.9 | 100% | CC | USA | CSA | M/R | Records | SPPC | |
| Mennen (1993) | 54 | 15.6 | 100% | CS | USA | CSA | M/R | Records | SPPC | |
| Moyer (1997) | 63 trauma, 131 control | 14 to 18 | 100% | CC | USA | CSA | M/R | Records | PHSCS | |
| Mwakanyamale (2019) | 1000 | 16.5 | 55% | CS | Tanzania | PM | M/R | Questionnaire | RSES | |
| Nguyen (2019) | 1149 | 16.1 | 64% | CS | Vietnam | PA, EA | M/R | Questionnaire | RSES | |
| Nguyen (2010) | 2591 | 15 | 52% | CS | Vietnam | EA, PA, CSA, Neglect | M/R | Questionnaire | RSES | |
| Oates (1994) | 130 | 9.7, 8.3 | 74% | CC | Australia | CSA | M/R | Records | Questionnaire, PHSCS | |
| Oates (1985) | 74 | 8.9 | 38% | CC | Australia | CA | M/R | Records | PHSCS | |
| O'Keefe (1998) | 939 | 16.9 | 49% | CS | USA | Violence | M/R | Modified CTS | RSES | |
| Orr (1985) | 22 | 12.9, 14.1 | 100% | CC | USA | CSA | M/R | Rec | Questionnaire | |
| Pantelewicz (2021) | 782 | 15.7 | 48% | CS | Poland | Abuse at home | M/R | Questionnaire |  | |
| Parent (2022) | 1055 | 15.3 | 72% | CS | Canada | Abuse in sport | M/R | Questionnaire | RSES | |
| Park (2018) | 1796 | 2nd and 3rd grade | 48% | L | Korea | PA, Neglect | M/R | Questionnaire | RSES | |
| Rana (2020) | 667 | 13 | 39% | CS | India | Bullying | M/R | Questionaire | RSES | |
| Reid-Russell (2022) | 240 | 12.4 | 42% | CS | USA | Abuse at home, CSA, PA | M/R | Questionnaire | Questionnaire | |
| Reyes (2008) | 63 trauma, 432 control | 10.8 | 64% | CC | USA | CSA | M/R | Records | SPPC | |
| Ronzon-Tirado (2022) | 1379 | 15.3 | 52% | CS | Spain | Abuse at home, relationship abuse | M/R | Questionnaire | RSES | |
| Rust (1991) | 25 trauma, 25 control | 12.5, 12.8 | 100% | CC | USA | CSA | M/R | Records | PHSCS | |
| Ryu (2023) | 2640 | 14 to 16 | 48% | CS | South Korea | Abuse at home, PA, EA | M/R | Questionnaire | Questionnaire | |
| Saigh (2008) | 92 trauma, 41 control | 14.4, 13.2, 12.5 | 43% 33%, 59% | CC | USA | Mixed trauma DSM-4 criteria | M/R | Records | PHSCS | |
| Salazar (2004) | 522 | 16.2 | 100% | CS | USA | Dating Violence | M/R | Questionnaire | RSES | |
| Sayar (2005) | 173 | 15.2 | 26% | CS | Turkey | PA | M/R | Questionnaire | RSES | |
| Scaicca (2023) | 728 | 14.4 | 50.4% | CS | Sweden | CSA | M/R | Questionnaire | RSES | |
| Scheer (2022) | 17112 | 15.6 | 43% | CS | USA | Bulling, CSA, Relationship Violence | M/R | Questionnaire | RSES | |
| Schlechter (2021) | 596 | 17.2 | 75% | CS | UK | Abuse at home | M/R | Questionnaire | RSES | |
| Sevenoaks (2022) | 158 | 13.7 | 53% | CS | South Afria | CSA, PA, EA, N | M/R | CTQ-SF | RSES | |
| Shah (2021) | 317 | 14.3 | 61% | CS | UAE | PA, EA, N | M/R | Questionnaire | RSES | |
| Shattnawi (2022) | 559 | 7 to 11 grade | 52% | CS | Jordan | EA, PAA, bullying, EN, PN, household violence | M/R | ACE | RSES | |
| Shen (2015) | 736 | 12 to 15 | 52% | CS | China | EA, CSA, PA, EN, PN | M/R | Questionnaire | RSES | |
| Skeen (2016) | 989 | 8.9 | 51% | L | Malawi & South Africa | Violence (domestic, comm-unity, physical, psychological) | M/R | Questionnaire | RSES | |
| Smith (2018) | 190 | 15.8 | 56% | CS | Canada | Cyber Dating Violence | M/R | Questionnaire | Questionnaire | |
| Soler (2012) | 722 | 14 to 18 | 64% | CS | Spain | Victimization (sexual), CM, | M/R | Questionnaire | RSES | |
| Stern (1995) | 84 | 9.3 | 74% | CC | Australia | CSA | M/R | Records | PHSC | |
| Sturkie (1987) | 40 trauma,54 control | NR | NR | CC | USA | PA, EA, PN, EN, CSA | M/R | Questionnaire | Questionnaire | |
| Suzuki (2015) | 342 | 13.5 | 56% | CS | Japan | PA, CSA, EA, EN | M/R | Questionnaire | RSES | |
| Swanston (1997) | 84 trauma,84 control | 15.1 | 74% | CC | Australia | CSA | M/R | Records | PHSCS | |
| Tocker (2017) | 27 trauma,27 control | 14.9 | 89% | CC | Finland | CSA | M/R | Records | RSES | |
| Tong (1987) | 90 | 11.5 | 76% | CC | Australia | CSA | M/R | Structured Interview | PHSCS | |
| Toth (1992) | 81 trauma,72 control | 7 to 12 | 40% | CC | USA | PA, PN | M/R | Records | Questionnaire | |
| Trickett (2011) | 303 trauma, 151 control | 10.9 | 50% | L | USA | EA, PA, CSA, Neglect | M/R | Records | Questionnaire | |
| Turner (2010) | 523 | 11 to 18 | 53% | L | USA | nonsexual maltreatment, sexual victimization, peer victimization | M/R | Questionnaire | modified RSES | |
| Vigil (2008) | 50 trauma, 31 control | 14.4 | 72% | CC | USA | Hurricane | Single | Questionnaire | RSES | |
| Wang (2020) | 9704 | 15.6 | NR | CS | China | EA, PA, CSA, EN, PN, CM | M/R | Questionnaire | RSES | |
| Weiler (2019) | 144 | 10.4 | 51% | RCT | USA | PA, CSA , exposure to community violence | M/R | Questionnaire | SPPC | |
| Wodarski (1990) | 69 trauma, 70 control | 12.5, 12.4, 11.9 | 59% 57% 26% | CC | USA | PA, N | M/R | Records | PHSCS | |
| Wonderlich (2001) | 40 | 10 to 15 | 100% | CC | USA | CSA | M/R | Questionnaire | PHSCS | |
| Wondie (2011) | 318 trauma, 318 control | NR | 100%, | CC | Ethiopia | CSA | M/R | Records | RSES | |
| Wu (2020) | 4790 | 16.8 | 49% | CS | China | Bullying Victimis-ation | M/R | Questionnaire | RSES | |
| Wu (2023) | 723 | 8 to 11 | 36% | CS | China | Bullying | M/R | Questionnaire | RSES | |
| Yoder (2005) | 501 | 14.9 | 52% | CS | USA | PA | M/R | Questionnaire | RSES | |
| Yoo (2021) | 3437 | 13 to 18 | 49% | L | South Korea | Cyberbullying | M/R | Questionnaire | RSES | |
| Yu (2021) | 1085 | 9.11 | 64.5% | CS | Chin | N | M/R | Questionnaire | RSES | |
| Zhang (2022) | 775 | 12.4 | 53% | CS | China | Mixed | M/R | ACE | RSES | |
| Zhou (2019) | 397 | 16.4 | 61% | CS | China | Earthquake | Single | Questionnaire | RSES | |
| Zhu (2020) | 18452 | 15.9 | 47% | CS | China | Child Victimisation | M/R | Questionnaire | RSES | |
| Zeller (2015) | 177 | 16.0 | 81% | CS | USA | EA, PA, CSA, EN, PN | M/R | Questionnaire | SPPA |  |

Note: Only first authors reported

NR=Not reported, CC= case-control, CS=cross-sectional, PA=physical abuse, EA = emotional abuse, CSA= sexual abuse, PN= physical neglect, EN= emotional neglect, PsM=psychological maltreatment, EM=emotional maltreatment, CM=childhood maltreatment PhM=physical maltreatment, N=neglect, CS=cross-sectional, L=Longitudinal; M/R= multiple or repeated trauma, RSES=Rosenberg Self-Esteem Scale, CAPA=Child and Adolescent Psychiatric Assessment, CF-SEI=Culture Free Self Esteem Scale CSEI=Coopersmith Self-Esteem Inventory, PHSCS=Piers-Harris children’s Self-Concept Scale, RCT=Randomised Controlled Trial; SDQ=Self-Description Questionnaire; SPPC=Self-Perception Profile for Children, SPPA=Self-Perception Profile for Adolescents, SEI= Self-Esteem Inventory, questionnaire= any other self-concept measure/trauma measure. *median

*Supplementary Material 4. Effect size and study participants for studies included in the meta-analysis*

| **Study (First Author, Year)** | **N** | **Effect Size** |
| --- | --- | --- |
| Ackard 2002 | 81247 | -0.15 |
| Aloba 2020 | 1337 | -0.3 |
| Arslan 2016 | 937 | -0.31 |
| Asgeirsdottir 2010 | 9113 | -0.16 |
| Ayhan 2023 | 270 | -0.24 |
| Baeg 2020 | 605 | -0.26 |
| Bagley 1992 | 369 | -0.4 |
| Bailey 2005 | 150 | -0.21 |
| BernardBonnin 2008 | 134 | -0.15 |
| Bolger 1998 | 107 | -0.09 |
| Brown 2019 | 5866 | -0.18 |
| Burack 2006 | 98 | -0.19 |
| Cecil 2001 | 249 | -0.13 |
| Cederbaum 2020 | 454 | -0.14 |
| Chang 2012 | 14 | -0.6 |
| Chen 2019 | 580 | -0.22 |
| Chen 2022a | 417 | -0.26 |
| Chen 2022b | 941 | -0.37 |
| Choi 2016 | 443 | -0.23 |
| Daniel 2016 | 540 | 0.06 |
| Deb 2016 | 370 | -0.08 |
| Dion 2021 | 227 | -0.13 |
| Dion 2022 | 1802 | -0.32 |
| Doku 2023 | 291 | -0.33 |
| Egan 1998 | 189 | 0.01 |
| Elliott 1990 | 34 | -0.04 |
| Esparza 1996 | 123 | -0.37 |
| Esparza-Del Villar 2022 | 526 | -0.07 |
| Flynn 2014 | 635 | -0.09 |
| Folayan 2020 | 1001 | -0.25 |
| Fonseca de Freitas 2022 | 2975 | -0.13 |
| Fu 2022 | 4313 | -0.31 |
| Garduno 2022 | 555 | -0.12 |
| Gauthier-Duchesne 2022 | 8194 | -0.14 |
| Genc 2018 | 3193 | -0.05 |
| German 1990 | 40 | -0.09 |
| Gesinde 2011 | 480 | -0.37 |
| GewirtzMeydan 2020 | 828 | -0.31 |
| Grayston 1992 | 69 | -0.38 |
| Greger 2016 | 1254 | -0.14 |
| Greger 2017 | 400 | -0.37 |
| Gunnlaugsson 2013 | 3515 | -0.29 |
| HajYahia 2002 | 1640 | -0.29 |
| Herd 2022 | 498 | -0.13 |
| Hibbard 1988 | 712 | 0.11 |
| Hibbard 1992 | 82 | -0.17 |
| Jankowiak 2021 | 1451 | -0.08 |
| Jezl 1996 | 257 | -0.1 |
| Johnson 2001 | 120 | -0.33 |
| Jonsson 2019 | 5715 | -0.06 |
| Ju 2018 | 2844 | -0.19 |
| Kaufman 1989 | 137 | -0.22 |
| Kim 2017 | 802 | -0.11 |
| Kim 2004 | 345 | -0.1 |
| Kim 2006 | 251 | -0.07 |
| Kim 2020 | 2844 | -0.10 |
| Kim 2021 | 4328 | -0.08 |
| Kocturk 2017 | 210 | -0.34 |
| Lam 2015 | 980 | -0.16 |
| Lau 2003 | 489 | -0.04 |
| Lee 2021 | 2321 | -0.15 |
| Leeson 2011 | 50 | -0.5 |
| Li 2009 | 1625 | -0.07 |
| Li 2023 | 1957 | -0.43 |
| Lim 2017 | 2351 | -0.29 |
| Lim 2020 | 1831 | -0.33 |
| Lin 2011 | 683 | -0.13 |
| Liu 2023 | 1064 | -0.26 |
| Luo 2020 | 1302 | -0.2 |
| Lynch 1998 | 322 | -0.003 |
| Ma 2014 | 366 | -0.26 |
| Malik 2016 | 400 | -0.35 |
| Mannarino 1989 | 169 | 0.02 |
| Maruyama 2022 | 1949 | -0.02 |
| Maskell 2013 | 1453 | -0.02 |
| Matejcek 1983 | 228 | -0.23 |
| Medora 1993 | 121 | -0.24 |
| Mennen 1994a | 1891 | -0.06 |
| Mennen 1994b | 1249 | -0.22 |
| Mennen 1993 | 54 | -0.27 |
| Moyer 1997 | 201 | -0.36 |
| Mwakanyamale 2019 | 1000 | -0.52 |
| Nguyen 2019 | 1149 | -0.15 |
| Nguyen 2010 | 2591 | -0.29 |
| Oates 1994 | 130 | -0.31 |
| Oates 1985 | 74 | -0.39 |
| OKeefe 1998 | 939 | -0.06 |
| Orr 1985 | 40 | -0.21 |
| Pantelewicz 2021 | 782 | -0.29 |
| Parent 2022 | 1055 | -0.14 |
| Park 2018 | 1796 | -0.12 |
| Rana 2020 | 632 | `-0.01 |
| Reid-Russell 2022 | 240 | -0.21 |
| Reyes 2008 | 495 | -0.1 |
| Ronzon-Tirado 2022 | 648 | -0.21 |
| Rust 1991 | 50 | -0.44 |
| Ryu 2023 | 2640 | -0.13 |
| Saigh 2008 | 133 | -0.23 |
| Salazar 2004 | 522 | -0.11 |
| Sayar 2005 | 173 | -0.24 |
| Scheer 2022 | 17112 | -0.47 |
| Schlechter 2021 | 596 | -0.3 |
| Sciacca 2023 | 728 | -0.11 |
| Sevenoaks 2022 | 158 | -0.15 |
| Shah 2021 | 317 | -.21 |
| Shattnawi 2022 | 559 | -0.09 |
| Shen 2015 | 736 | -0.03 |
| Skeen 2016 | 989 | -0.13 |
| Smith 2018 | 190 | -0.07 |
| Soler 2012 | 722 | -0.08 |
| Stern 1995 | 84 | -0.39 |
| Sturkie 1987 | 94 | -0.19 |
| Suzuki 2015 | 342 | -0.13 |
| Swanston 1997 | 168 | -0.37 |
| Tocker 2017 | 54 | -0.36 |
| Tong 1987 | 90 | -0.33 |
| Toth 1992 | 153 | -0.21 |
| Trickett 2011 | 454 | -0.12 |
| Turner 2010 | 523 | -0.17 |
| Vigil 2008 | 81 | -0.3 |
| Wang 2020 | 9704 | -0.17 |
| Weiler 2019 | 144 | -0.27 |
| Wodarski 1990 | 139 | -0.17 |
| Wonderlich 2001 | 40 | -0.2 |
| Wondie 2011 | 636 | -0.32 |
| Wu 2020 | 4790 | -0.18 |
| Wu 2023 | 723 | -0.14 |
| Yoder 2005 | 501 | -0.21 |
| Yoo 2021 | 3437 | -0.03 |
| Yu 2021 | 1085 | -0.58 |
| Zeller 2015 | 177 | -0.21 |
| Zhang 2022 | 775 | -0.28 |
| Zhou 2019 | 397 | -0.05 |
| Zhu 2020 | 13370 | -0.11 |

Supplementary Material 5. Forest plot for CSA studies.


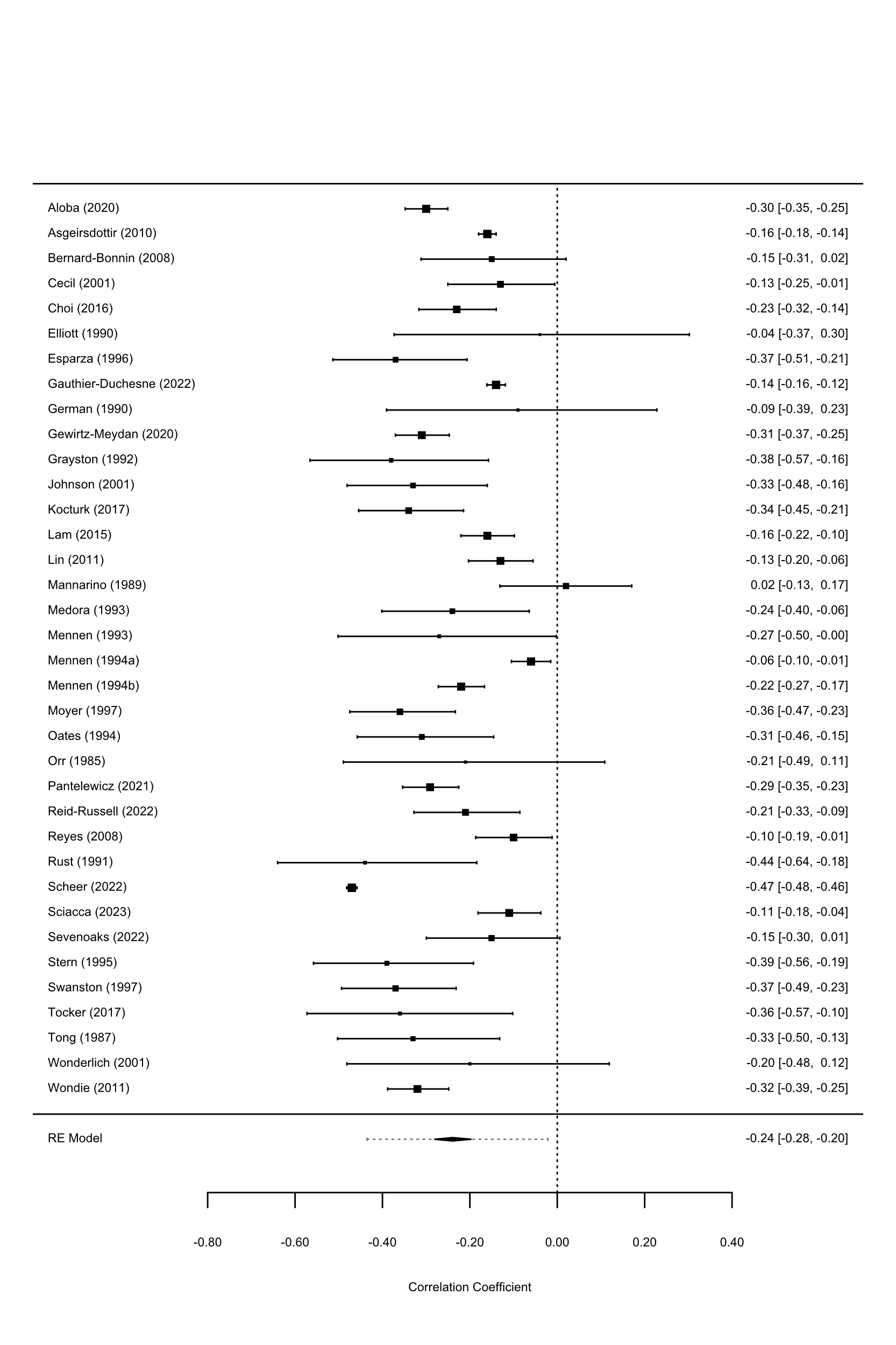


Supplementary Material 6. Forest plot for mixed trauma exposure studies.


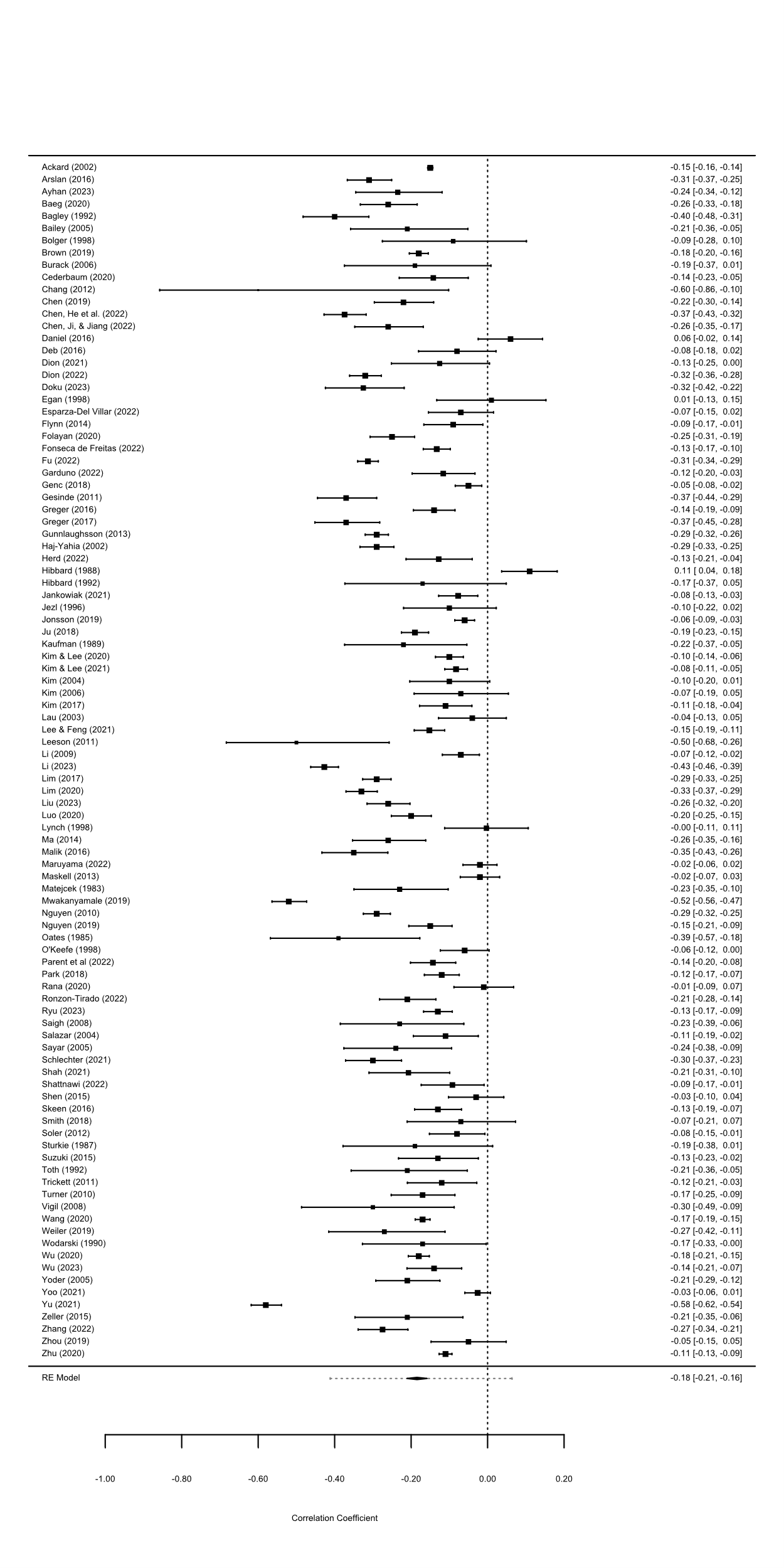


Supplementary Material 7. Funnel plot.


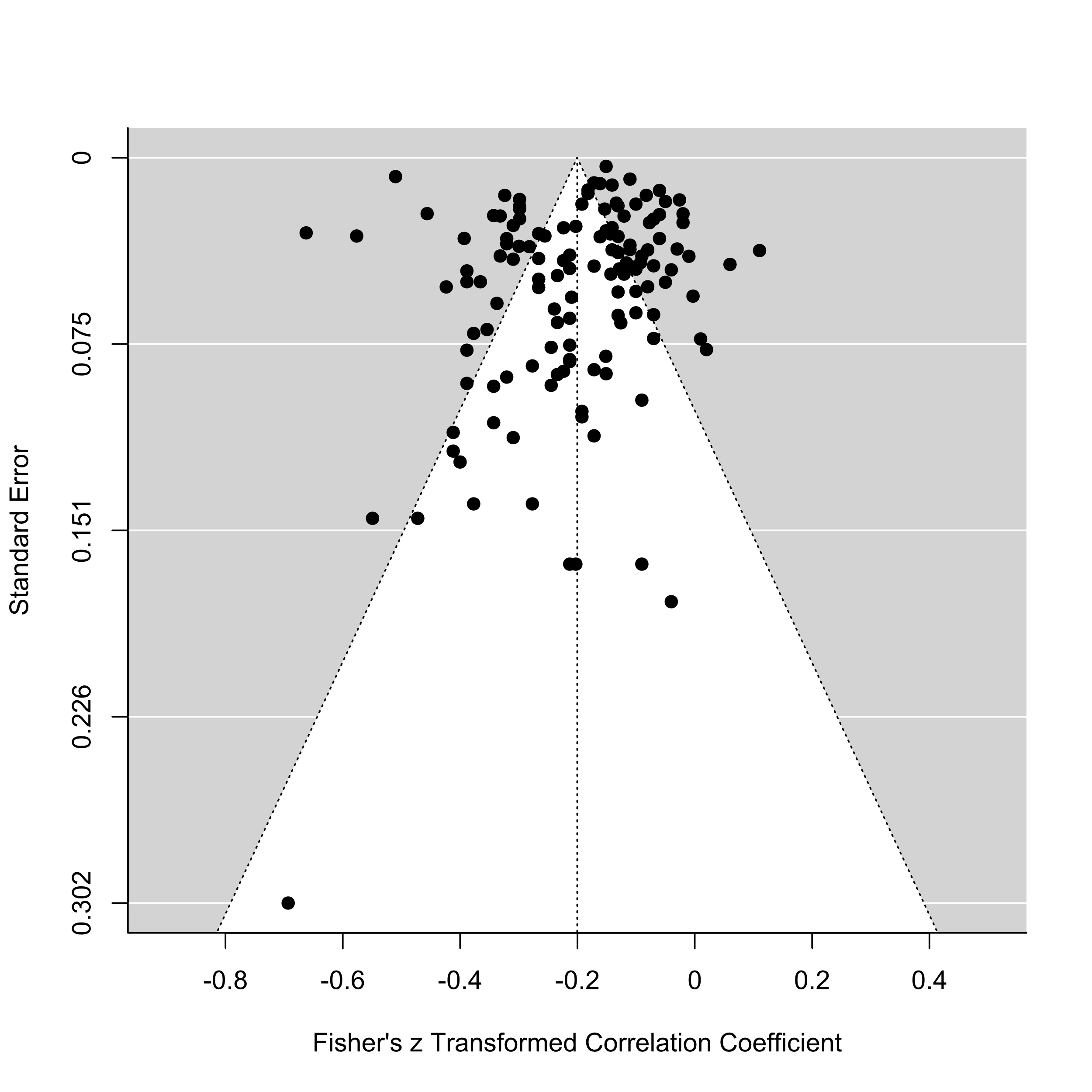

Supplement: Supplementary file 1 — Supplementary file1 (DOCX 1312 kb) [file 10567_2024_472_MOESM1_ESM.docx]
